# Nearly unbiased estimator of contemporary effective mother size using within-cohort maternal sibling pairs incorporating parental and nonparental reproductive variations

**DOI:** 10.1038/s41437-019-0271-6

**Published:** 2019-10-02

**Authors:** Tetsuya Akita

**Affiliations:** National Research Institute of Fisheries Science, Japan Fisheries Research and Education Agency, 2-12-4 Fukuura, Kanazawa, Yokohama, 236-8648 Kanagawa Japan

**Keywords:** Molecular ecology, Population genetics

## Abstract

In this study, we developed a nearly unbiased estimator of contemporary effective mother size in a population, which is based on a known maternal half-sibling relationship found within the same cohort. Our method allows for variance of the average number of offspring per mother (i.e., parental variation, such as age-specific fecundity) and variance of the number of offspring among mothers with identical reproductive potential (i.e., nonparental variation, such as family-correlated survivorship). We also developed estimators of the variance and coefficient of variation of contemporary effective mother size and qualitatively evaluated the performance of the estimators by running an individual-based model. Our results provide guidance for (i) a sample size to ensure the required accuracy and precision when the order of effective mother size is available and (ii) a degree of uncertainty regarding the estimated effective mother size when information about the size is unavailable. To the best of our knowledge, this is the first report to demonstrate the derivation of a nearly unbiased estimator of effective population size; however, its current application is limited to effective mother size and situations, in which the sample size is not particularly small and maternal half-sibling relationships can be detected without error. The results of this study demonstrate the usefulness of a sibship assignment method for estimating effective population size; in addition, they have the potential to greatly widen the scope of genetic monitoring, especially in the situation of small sample size.

## Introduction

Contemporary effective population size, which is sensitive to ecological time-scale events, has become recognized as an informative parameter in a focus population, especially in the context of conservation biology and wildlife management (Luikart et al. 2010). There are several methods for estimating contemporary effective population size from genetic markers, such as the temporal method (Nei and Tajima 1981), heterozygote excess method (Pudovkin et al. 1996), molecular coancestry method (Nomura 2008), linkage-disequilibrium method (Waples 2006), and kinship assignment method (Wang 2009). At present, it is known that values estimated by these methods display large uncertainties and/or biases under conditions, such as small sample size, small marker numbers, and large effective population size; thus, a widely applicable method is required (Wang et al. 2016; Marandel et al. 2018).

Owing to rapid developments in genotyping technology, a large number of genetic markers, including thousands of genome-wide single nucleotide polymorphisms, have become available for analyzing population structure and demography. As a result, a more accurate estimation of contemporary effective population size can be obtained by, for example, more accurately assigned kinships (Wang et al. 2016). In addition, the recently developed theory of estimation of absolute adult number, which is based on sampled kinship pairs and known as the close-kin mark-recapture (CKMR) method (Bravington et al. 2016a, b; Skaug 2017; Hillary et al. 2018), makes it possible to use a full-sibling (FS) or half-sibling (HS) pair; this involves many more DNA markers for detection than a parent–offspring pair. It should be noted that the CKMR method is designed to minimize the effect of reproductive variance originating from unmodeled covariates, such as avoiding the use of sibling pairs sampled from the same cohorts; meanwhile, reproductive variance strongly affects the estimation of contemporary effective population size.

Reproductive variance has two components. The first component is variation in age, size, and other factors, which affects average fecundity and originates from differences in life-history parameters (Felsenstein 1971). For example, in the case of teleost species that have a long life span, the number of eggs produced by a mother (i.e., annual fecundity) is determined by her body size; thus, there is considerable variation in reproduction among mothers. The second component is variation in reproduction among parents of the same age or size. An extreme case reflecting this variation is referred to as the “Sweepstakes Reproductive Success (Hedgecock and Pudovkin 2011),” in which only several families reproduce successfully. This phenomenon has received much attention not only for elucidating the ecology of species that display highly variable early life mortality (i.e., type-III life history) but also for providing an opportunity to test the applicability of the multiple-merger coalescent model, a recently developed theory in population genetics (Tellier and Lemaire 2014; Eldon et al. 2016). Addressing the two aforementioned types of variance together can provide insights for interpreting estimated values of effective population size.

In this paper, we propose a new method for estimating the contemporary effective mother size in a population. This approach is based on the number of maternal HS (MHS) pairs found within the same cohort and on modeling that explicitly incorporates overdispersed reproduction, assuming that kinships are genetically detected without any error. Our model partitions reproductive variance into two types of variations: (i) age- or size-specific differences in mean fecundity (referred to as “parental variation”) and (ii) unequal contributions by mothers of the same age or size to the number of offspring at sampling (referred to as “nonparental variation”) First, we formulate the distribution of offspring number under the two types of variations. Second, we analytically derive the probability that two randomly selected individuals found in the same cohort share an MHS relationship. Third, we determine a nearly unbiased estimator of contemporary effective mother size and its relative estimators. Finally, we investigate the performance of the estimators by running an individual-based model. Our modeling framework may be applied to diverse animal species; however, the description of the model focuses on fish species, which are currently the best candidate target of our proposed method.

## Theory

Main symbols used in this paper are summarized in Table 1.

**Table 1:** List of mathematical symbols in main text

*n*	Sample number of offspring
*n*_pair_	Number of pairs in a sample (=_*n*_C_2_)
*N*	Number of mothers in the population when sampled offspring are born
*N*_e_	Effective number of mothers in the population
*ϕ*	Overdispersion parameter under negative binomial reproduction
*λ*_*i*_	Expected number of surviving offspring of mother *i* at sampling
*f*(*λ*)	Frequency of *λ* for all mothers
*k*_*i*_	Number of surviving offspring born to mother *i*
*H*	Number of maternal half-sibling pairs found in samples
*π*	Probability that a randomly selected pair (two offspring) share a maternal half-sibling relationship
*c*	Combined effect of deviation from the Poisson (=(1 + *ϕ*^−1^)${\Bbb E}$[*λ*^2^]/${\Bbb E}$[*λ*]^2^)
$\hat N_{{\mathrm{e}},{\mathrm{0}}}$	Moment estimator of *N*_e_
$\hat N_{{\mathrm{e}},{\mathrm{1}}}$	Nearly unbiased estimator of *N*_e_
$\hat N_{{\mathrm{e}},{\mathrm{TM}}}$	Moment estimator of *N*_e_ by the temporal method
$\hat v$	Estimator of ${\Bbb V}[\hat N_{{\mathrm{e}},{\mathrm{1}}}]$
$\widehat {cv}$	Estimator of ${\Bbb C}{\Bbb V}[\hat N_{{\mathrm{e}},{\mathrm{1}}}]$
*b*_mean_	Bias of $\hat N_{{\mathrm{e}},{\mathrm{1}}}$
*b*_var_	Bias of $\hat v$

### Hypothetical population and sampling scheme

Here, we suppose that there is a hypothetical population consisting of *N* mothers and that there is no population subdivision or spatial structure. In this paper, a mature female is referred to as a mother even if she does not produce offspring. For the detection of MHS pairs, *n* offspring within the same cohort are simultaneously and randomly sampled in the population. For mathematical tractability, we assume that there is only one spawning ground in which the mothers remain for the entire spawning season.

In our modeling framework, if an MHS pair also shares a paternal HS (PHS) relationship, the pair is considered to be an MHS pair (i.e., the FS relationship is assigned as MHS relationship). The technical difficulties of distinguishing an MHS pair from a PHS pair are addressed in the “Discussion” section.

### Reproductive potential and its variation (parental variation)

Here, we introduce the concept of the reproductive potential of mother *i* (*i* = 1, 2, …, *N*), which is defined as the expected number of surviving offspring at sampling time, denoted by *λ*_*i*_. The reproductive potential is determined by several factors, including the mother’s age, weight, residence time on the spawning ground, and it is allowed to vary across mothers. In this study, this variation is referred to as parental variation. It should be noted that the magnitude of this parameter (*λ*_*i*_) includes information about the survival rate of the offspring, the number of days after egg hatching, and the egg number; this implies that the parameter reflects the sample timing. It should also be noted that the modeling framework does not depend on whether the reproductive potential is heritable or not.

### Nonparental variation

**Fig. 1:**
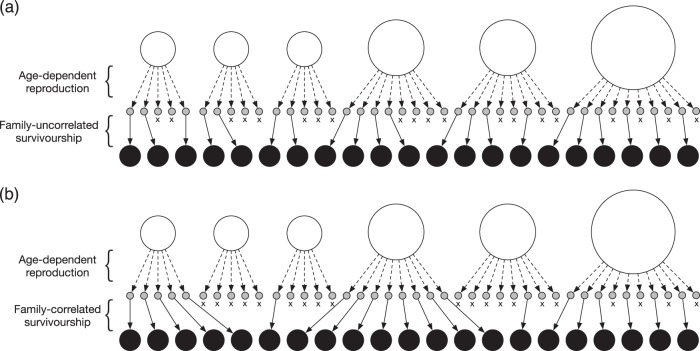
Example of relationships between mothers and their offspring number for only parental variation (**a**) and both parental- and nonparental variation (**b**). *N* = 6 and $\mathop {\sum}_i^N {k_i} = 21$. Open, gray, and black circles represent mothers, their eggs, and their offspring, respectively. The area of an open circle indicates the degree of reproductive potential of each mother (i.e., *λ*_*i*_). Dotted and thin arrows show mother–egg and egg–offspring relationships, respectively. The x symbol indicates a failure to survive at sampling

In addition to parental variation, the variation in reproduction among mothers with the same reproductive potential, referred to here as nonparental variation, is also incorporated into the model, resulting in a large variation in the fertility of the mothers. As the magnitude of the variance increases, the number of successful mothers producing offspring that avoid early life mortality decreases, leading to a situation in which offspring derived from the same mother has highly correlated early life survival probabilities. This situation requires careful consideration of the probability that two offspring share an MHS relationship. Figure 1 presents a schematic representation of the effects of such family-correlated survival on kinship relationships in a population, which are exemplified in iteroparous teleost species. Older mothers are more likely to produce a larger number of offspring, as annual fecundity (i.e., number of eggs, represented by a gray circle) increases with age. However, due to family-correlated survivorship after eggs hatching, the probability that two offspring (i.e., at the larva or juvenile stage, represented by a closed circle) have an MHS relationship is higher (e.g., 53 MHS pairs in Fig. 1b) than in a situation with independent survival (e.g., 32 MHS pairs in Fig. 1a). In other words, MHS pairs have significantly higher or lower collective chances for survival. In addition to family-correlated survivorship, the effects of mating behavior are also incorporated into nonparental variation, such as competition for males/females and correlation between mating opportunities of mother and her offspring number. Nonparental variation may occasionally overshadow the effect of parental variation; however, the average number of offspring per mother is higher for an older mother because the probability of being a successful mother driven by nonparental variation is not biased among mothers.

### Distribution of offspring number

In attempting to incorporate both parental and nonparental variation, it is useful to employ a highly skewed distribution of offspring number. In this study, we use a negative binomial distribution, which is applicable to deviation from the Poisson variance (i.e., overdispersed offspring number with a variance greater than the mean).

Let *k*_*i*_ be the number of surviving offspring of mother *i* at sampling. Given the expected number of offspring *λ*_*i*_, *k*_*i*_ is assumed to follow a negative binomial distribution by a conventional parametrization,

1 $${\mathrm{Pr}}[k_i|\lambda _i] = \frac{{\Gamma [k_i\,+\,\phi ]}}{{k_i!\Gamma [\phi ]}}\left( {\frac{{\lambda _i}}{{\phi\,+\,\lambda _i}}} \right)^{k_i}\left( {\frac{\phi }{{\phi\,+\,\lambda _i}}} \right)^\phi ,$$

where *ϕ* (>0) is the overdispersion parameter describing the degree of nonparental variation (Akita 2018). At present, *ϕ* is assumed to be constant across mothers, whereas the expected number of surviving offspring (*λ*_*i*_) is variable across mothers. The mean and variance of this distribution are *λ*_*i*_ and $\lambda _i + \lambda _i^2/\phi$, respectively. In the limit of infinite *ϕ*, this distribution becomes a Poisson distribution as follows:

2 $$\mathop {{\lim }}\limits_{\phi \to \infty } {\mathrm{Pr}}[k_i|\lambda _i] = \frac{{\lambda _i^{k_i}{\mathrm{e}}^{ - \lambda _i}}}{{k_i!}}.$$

We assume that *λ*_*i*_ is independent and identically distributed with a density function *f*(*λ*), which produces parental variation. The shape of the density function is often complex but may be described by information such as the mother’s weight composition in the population. The specific form of *f*(*λ*) is provided in Appendix A and is used for verifying the theory developed in this paper. As explained in the next subsection, the theory does not require this specific form; it only requires the ratio of the second moment to the squared first moment (i.e., ${\Bbb E}$[*λ*^2^]/${\Bbb E}$[*λ*]^2^).

### MHS probability among randomly selected individuals

We have derived the approximate probability that two offspring share an MHS relationship with an arbitrary mother (denoted by *π*) as follows:

3 $$\pi\,\approx\,\frac{c}{{N\,+ c\,- 1}},$$

where

$$c = (1 + \phi ^{ - 1})\frac{{{\Bbb E}[\lambda ^2]}}{{{\Bbb E}[\lambda ]^2}}.$$

The details of the derivation is provided in Appendix B. Equation (3) explicitly contains the two variations (i.e., parental variation and nonparental variation) that determine the degree of deviation from the Poisson distribution. When *λ* is constant across mothers, ${\Bbb E}$[*λ*^2^] equals ${\Bbb E}$[*λ*]^2^ and then *π* becomes (1 + *ϕ*^−1^)/(*N* + *ϕ*^−1^), which appears in Eq. (7) in Akita (2018). In addition, as *ϕ*→∞, (1 + *ϕ*^−1^)/(*N* + *ϕ*^−1^) converges to 1/*N*, which corresponds to the Poisson variance of *k*_*i*_ for all mothers in a population. The effect of the two factors causing a deviation from the Poisson distribution can be combined as parameter *c* (≥1). Hereafter, “overdispersion” is referred to as the distribution of the number of offspring resulting from this combined effect.

When *N* is provided, *π* increases with an increase in *c*, suggesting that a randomly selected pair is more likely to share an MHS relationship under greater overdispersion. Figure S1a–d (Supplementary Information) illustrates the theoretical curve and the simulation results of *π* with *N* = 100 and 10,000 as a function of *ϕ* or ${\Bbb E}$[*λ*^2^]/${\Bbb E}$[*λ*]^2^. This figure demonstrates that the approximation in Eq. (3) works well for the investigated function *f*(*λ*).

### Skewed offspring distribution by parental and nonparental variation

**Fig. 2:**
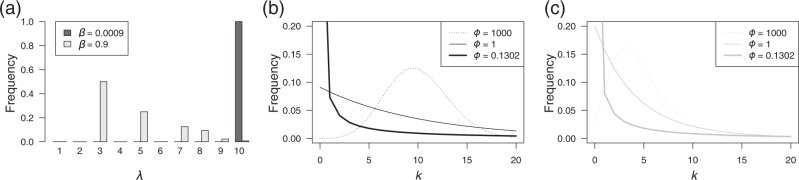
**a** Histogram of *f*(*λ*) assuming fish species with a relatively low *β* (denoted by black bar, ${\Bbb E}$[*λ*^2^]/${\Bbb E}$[*λ*]^2^ = 1.0000) and high *β* (denoted by gray bar, ${\Bbb E}$[*λ*^2^]/${\Bbb E}$[*λ*]^2^ = 1.1519). **b**, **c** Marginal distribution of *k* for several values of *ϕ* (see legend). **b** *β* = 0.0009; Pr[*k* = 0] with *ϕ* = 0.1302 equals 0.57. **c** *β* = 0.9; Pr[*k* = 0] with *ϕ* = 0.1302 equals 0.63. Details of *f*(*λ*) are provided in Appendix A

For illustrative purposes, we demonstrate how parental and nonparental variation skew the offspring distribution in an age-structured population. First, we explore the case in which parental variation is moderately observed and the case in which it is scarcely observed. The cases can be controlled by changing the parameters affecting the shape of *f*(*λ*). Suppose that the mean fecundity of a mother depends on her age, which can be considered as the reproductive potential. Let *λ*_*a*_ be mean fecundity, which is a function of age (denoted by *a*). Assuming that individual fecundity is proportional to weight and using the von Bertalanffy growth equation for body weight, *λ*_*a*_ is explicitly described as a function of age as follows:

4 $$\lambda _a \propto (1 - {\mathrm{exp}}[ - \kappa (a - a_0)])^\beta ,$$

where *κ*, *a*_0_, and *β* are conventionally used parameters in the von Bertalanffy equation and represent the growth rate, the adjuster of the equation for the initial size of the animal, and the allometric growth parameter, respectively. This relationship indicates that the age distribution generates the variation of *λ*_*a*_. Given the age distribution, the variation of *f*(*λ*) increases with *β*; meanwhile, when *β* goes to zero, the variation of *f*(*λ*) vanishes. Figure 2a presents a histogram of *f*(*λ*) for the two cases.

Next, we explore *f*(*λ*) with several combinations of the magnitude of parental and nonparental variations. Figure 2b, c illustrates the offspring distribution with a relatively low *β* and a moderate *β*, respectively. If both parental and nonparental variations are very small, *k* has as a Poisson distribution (dotted line in Fig. 2b), as noted above. When there is no parental variation, nonparental variation skews the distribution of *k* (thin and bold lines in Fig. 2b), and vice versa (dotted line in Fig. 2c). In this study, we selected parameter *c* = (1 + *ϕ*^−1^)${\Bbb E}$[*λ*^2^]/${\Bbb E}$[*λ*]^2^ to be 1 and 10 for comparison with the results. These two values represent two extreme cases and can be derived from the parameter set (*ϕ*, *β*) = (1000, 0.0009) (dotted line in Fig. 2b) and (0.1302, 0.9) (bold line in Fig. 2c), respectively. It should be noted that in the latter case, the offspring distribution is highly skewed: 63% of mothers cannot produce surviving offspring at sampling, and 6% of mothers produce more than 20 offspring (${\Bbb E}$[*λ*] = 4.5 and ${\Bbb V}$[*λ*] = 157.5). Other parameter values used in *f*(*λ*) are provided in Appendix A.

### Effective mother size and census size

We have defined the effective mother size as follows:

$$N_{\mathrm{e}} = \frac{1}{\pi }$$

5 $$= \frac{{N - 1}}{c} + 1.$$

This definition is similar to the inbreeding effective population size (Nordborg and Krone 2002), as the probability of sharing an MHS relationship (*π*) is identical to the probability that two individuals share a mother in the previous breeding season. It should be noted that when sampling from a single cohort in a population with overlapping generations, the effective mother size in our definition corresponds to the effective breeding mother size, which produces a single cohort.

One might ask whether the proposed effective size is consistent with previous work, such as the drift-based effective population size. When *λ* is constant, the drift-based effective population size is provided as follows:

$$N_{\mathrm{e}} = \frac{{\lambda ^2}}{{{\Bbb V}[k]}}N$$

6 $$= \frac{N}{{\lambda ^{ - 1}\,+\,c - 1}},$$

where ${\Bbb V}$[*k*] = *λ* + *λ*^2^/*ϕ* and *c* = 1 + *ϕ*^−1^. This derivation is based on the natural extension of the existing approach (e.g., Gillespie 2004), relaxing the assumption that mean size of a population does not change (i.e., *λ* = 1). The formulation is similar to the proposed effective size in Eq. (5), but not consistent except for the case of *λ* = 1.

Using Eq. (5), the ratio of the effective mother size to census size can be written as follows:

$$\frac{{N_{\mathrm{e}}}}{N} = \frac{1}{c} + \frac{1}{N}\left( {1 - \frac{1}{c}} \right)$$

7 $$\approx \frac{1}{c},$$

where *N* ≫ 1 is assumed for the purpose of approximation.

### Statistical properties of MHS pair number

In this subsection, given the unconditional probability that two offspring share an MHS relationship (Eq. (3)), we consider the distribution of the number of MHS pairs and its statistical properties. Let *H* be the number of MHS pairs found in an offspring sample of size *n*. First, we derive the approximate distribution of *H* for a situation in which overdispersion does not exist (i.e., *c* = 1). Second, we evaluate whether the derived distribution of *H* for the nonoverdispersed case is applicable to the overdispersed case (i.e., *c* > 1).

If overdispersion does not exist (i.e., *c* = 1), drawing an MHS pair from a randomly selected pair in a sample is considered a Bernoulli trial. Thus, *H* follows a hypergeometric distribution, which is a function of the sample size of the offspring, the total number of offspring in the population, and the total number of MHS pairs in the population. However, in the setting of this study, the latter two components are random variables, thus creating a complex situation for deriving the exact formulation (Akita 2018). Therefore, assuming that the total number of MHS pairs in the population is much higher than the number of pairs in a sample $_{{\sum} k }{\mathrm{C}}_2 \gg _n{\mathrm{C}}_2$ the distribution is approximated by a binomial form as follows:

8 $${\mathrm{Pr}}[H = h|n_{{\mathrm{pair}}},\pi ] = \left( \begin{array}{c}n_{{\mathrm{pair}}}\\ h\end{array} \right)\pi ^h(1 - \pi )^{n_{{\mathrm{pair}}} - h},$$

where *n*_pair_ is the number of pairs in a sample (=_*n*_C_2_). For practical purposes, the condition $_{{\sum} k }{\mathrm{C}}_2 \gg _n{\mathrm{C}}_2$ may be acceptable. The theoretical expectation of *H* is

9 $${\Bbb E}[H] = n_{{\mathrm{pair}}}\pi ,$$

and the variance is

10 $${\Bbb V}[H] = n_{{\mathrm{pair}}}\pi (1 - \pi ).$$

Figure S2a, b (Supplementary Information) illustrates the accuracy of the theoretical prediction for the expectation and the variance of *H* under the Poisson variance as a function of *n*, respectively. For the investigated parameter, the prediction is demonstrated to be highly accurate.

If overdispersion exists (i.e., *c* > 1), drawing an MHS pair is no longer a Bernoulli trial. For example, an individual that is born to a relatively successful mother has a greater probability of an MHS relationship with other individuals. Therefore, a hypergeometric/binomial form is not appropriate for the distribution of *H*. As illustrated in Fig. S2d (Supplementary Information), the binomial variance (Eq. (10)) is downwardly biased from the observed variance of *H* when *n* increases. The theoretical evaluation is relatively complex and is left for future research. However, for the investigated parameter set, the expected value is well approximated by Eq. (9) (Fig. S2c in Supplementary Information), assuming independent comparisons. The rationale may be that the MHS probability in a pair, *π* (Eq. (3)), includes the effect of overdispersion. Next, on the basis of an accurate approximation of ${\Bbb E}$[*H*] in the case of overdispersion, we provide the estimator of *N*_e_ from the observed number of MHS pairs in a sample.

### Moment estimator of ***N***_e_ from observed number of MHS pairs

By removing *π* in Eqs. (5) and (9), *N*_e_ can be written as a function of *c*, *n*_pair_, and ${\Bbb E}$[*H*]. The observed number of MHS pairs in a sample is defined by *H*_obs_, and ${\Bbb E}$[*H*] is replaced by *H*_obs_, generating the moment estimator of *N*_e_:

11 $$\hat N_{{\mathrm{e}},{\mathrm{0}}} = \frac{{n_{{\mathrm{pair}}}}}{{H_{{\mathrm{obs}}}}}.$$

In this paper, a “hat” indicates the estimator of a variable. This relationship can be written as follows:

12 $$\widehat {\left( {\frac{{N - 1}}{c}} \right)} = \frac{{n_{{\mathrm{pair}}}}}{{H_{{\mathrm{obs}}}}} - 1,$$

indicating that *N* and *c* cannot be estimated simultaneously from the number of observed MHS pairs.

Assuming that *H* follows a binomial distribution, the estimator corresponds to the maximum likelihood estimator of *N*_0_ (see Appendix C). There are two drawbacks to using this estimator. First, the value of $\hat N_{{\mathrm{e}},{\mathrm{0}}}$ becomes inflated when no MHS pairs are observed in a sample (i.e., *H*_obs_ = 0). This leads to a situation in which an individual-based model frequently generating zero MHS pairs is not available for statistical evaluation. Second, even if an MHS pair is detected in a sample, it is likely that $\hat N_{{\mathrm{e}},{\mathrm{0}}}$ is strongly biased (see Appendix C). Therefore, an improved estimator is necessary for the purpose of appropriate evaluation and higher accuracy for a wide parameter range.

### Nearly unbiased estimator of *N*_e_

We have derived an alternative estimator of *N*_e_ (denoted by $\hat N_{{\mathrm{e}},{\mathrm{1}}}$) as follows:

13 $$\hat N_{{\mathrm{e}},{\mathrm{1}}}\,=\,\frac{{n_{{\mathrm{pair}}}\,+\,1}}{{H_{{\mathrm{obs}}}\,+\,1}}.$$

The derivation process is similar to that of the nearly unbiased estimator of adult number in a population using the mark-recapture method (Chapman 1951), which is based on the idea that the observation of 1/(*H* + 1) approximately provides a linear estimator of *N*_e_ (see Appendix D). The bias of $\hat N_{{\mathrm{e}},{\mathrm{1}}}$ is defined by *b*_mean_, which is given by

$$b_{{\mathrm{mean}}} = {\Bbb E}[\hat N_{{\mathrm{e}},{\mathrm{1}}}] - N_{\mathrm{e}}$$

14 $$= - N_{\mathrm{e}}(1 - N_{\mathrm{e}}^{ - 1})^{n_{{\mathrm{pair}}}\,+\,1}.$$

It should be noted that $\hat N_{{\mathrm{e}},{\mathrm{1}}}$ is downwardly biased; however, this bias may be ignored for a wider range of parameters than $\hat N_{{\mathrm{e}},{\mathrm{0}}}$ (see details in the “Results” section), which allows $\hat N_{{\mathrm{e}},{\mathrm{1}}}$ to be called a nearly unbiased estimator.

We also determined the estimator of ${\Bbb V}[\hat N_{{\mathrm{e}},{\mathrm{1}}}]$, given by

15 $$\hat v\,=\,\frac{{(n_{{\mathrm{pair}}}\,+\,1)(n_{{\mathrm{pair}}} - H_{{\mathrm{obs}}})}}{{(H_{{\mathrm{obs}}}\,+\,1)^2(H_{{\mathrm{obs}}}\,+\,2)}}.$$

The derivation process is similar to that in Seber (1970) (see Appendix E for details). The bias of $\hat v$ is defined by *b*_var_, which is given by

$$b_{{\mathrm{var}}} = {\Bbb E}[v] - {\Bbb V}[\hat N_{{\mathrm{e}},{\mathrm{1}}}]$$

$$= N_{\mathrm{e}}^2\left( {(1 - N_{\mathrm{e}}^{ - 1})^{n_{{\mathrm{pair}}}\,+\,2}} \right.$$

16 $$\left. { + \left( {(n_{{\mathrm{pair}}}\,+\,2)N_{\mathrm{e}}^{ - 1} - 2} \right)(1 - N_{\mathrm{e}}^{ - 1})^{n_{{\mathrm{pair}}}\,+\,1}\,+\,(1 - N_{\mathrm{e}}^{ - 1})^{2(n_{{\mathrm{pair}}}\,+\,1)}} \right).$$

Finally, we consider the estimator of the coefficient of variation of $\hat N_{{\mathrm{e}},{\mathrm{1}}}$. A method similar to the derivation of $\hat v$ (i.e., searching for a formula such that its expectation approximates ${\Bbb C}{\Bbb V}[\hat N_{{\mathrm{e}},{\mathrm{1}}}]$) was overly complex for the estimator; instead, using Eqs. (13) and (15), we defined the estimator as follows:

$$\widehat {cv} = \frac{{\sqrt {\hat v} }}{{\hat N_{{\mathrm{e}},{\mathrm{1}}}}}$$

17 $$= \sqrt {\frac{{n_{{\mathrm{pair}}} - H_{{\mathrm{obs}}}}}{{(n_{{\mathrm{pair}}}\,+\,1)(H_{{\mathrm{obs}}}\,+\,2)}}} ,$$

Roughly speaking, $\widehat {cv}$ is approximated by $\sqrt {1/(H_{{\mathrm{obs}}} + 2)}$ because *n*_pair_ ≫ *H*_obs_, which is similar to an approximate lower bound on the coefficient of variation of $\hat N$, as presented in Bravington et al. (2016b).

### Individual-based model

To evaluate the performance of the estimators ($\hat N_{{\mathrm{e}},{\mathrm{1}}}$, $\hat v$, and $\widehat {cv}$), we developed an individual-based model that tracks kinship relationships. The population structure was assumed to be identical to that in the development of the estimators. The population consisted of mothers and their offspring and was assumed to follow a Poisson or negative binomial reproduction. The expected number of surviving offspring of a mother followed the density distribution *f*(*λ*), which was deterministically specified under stable age structure (see Appendix A). It should be noted that the overdispersion parameter (*c*) was calculated from *ϕ* and *f*(*λ*). Each offspring retained the ID of its mother, making it possible to trace an MHS relationship.

Given a parameter set (*N*, *n*, *ϕ*, and parameters that determine *f*(*λ*)), we simulated a population history in which *N* mothers generated offspring; this process was repeated 100 times. For each history, the sampling process was repeated 1000 times, acquiring 100,000 data points that were used to construct the distribution of $\hat N_{{\mathrm{e}},{\mathrm{1}}}$, $\hat v$, and $\widehat {cv}$ for each parameter set. *N*_e_ was calculated from *N* and *c* (Eq. (5)).

### Temporal method

To compare the performance between our method and other existing methods, we considered the temporal method, which is based on a moment estimator (Nei and Tajima 1981). The temporal method relies on the temporal changes in allele frequency over time, as information for estimating *N*_e_. To calculate the estimator of *N*_e_ by the temporal method, simulations were independently run and analyzed.

We evaluated the performance of the temporal method estimator on data simulated under the Wright-Fisher model for a haploid population. For a given *N*_e_, the frequency trajectory of 500 independent loci was simulated. For each locus, the maximum number of alleles was set to 10 and initial frequencies of those alleles were fixed to 0.1 at generations 0. Two samples of *n* individuals were each randomly taken at generation 0 and 9 from the offspring gene pool (i.e., sampling without replacement). For each combination of parameters (*N*_e_, *n*), we run 100,000 replicates and obtained the estimator of *N*_e_ (denoted by $\hat N_{{\mathrm{e}},{\mathrm{TM}}}$) for each replicate. For comparisons, we set an equal sample size at one time for both methods, although the total sampling size of the temporal method was twice that of our method. In the current comparison, we did not consider the case of overdispersion (e.g., *c* > 1) because an estimation of an extra parameter is needed (see Kitada et al. 2000) and the comparison of the case goes beyond the scope of this work.

## Results

We evaluated the performance of the estimators ($\hat N_{{\mathrm{e}},{\mathrm{1}}}$, $\hat v$, and $\widehat {cv}$) for a situation in which the number of mothers, *N*, and the combined effect of deviation from the Poisson, *c*, were unknown. The parameter values were changed for *N* (100, 1000, 10,000, and 100,000) and *c* (1 and 10). We primarily addressed the number of samples (*n*) required to provide adequate accuracy and precision under a given parameter set (*N* and *c*).

### Comparison of the bias of $\hat N_{{\mathrm{e}},{\mathrm{1}}}$ with that of $\hat N_{{\mathrm{e}},{\mathrm{0}}}$

**Fig. 3:**
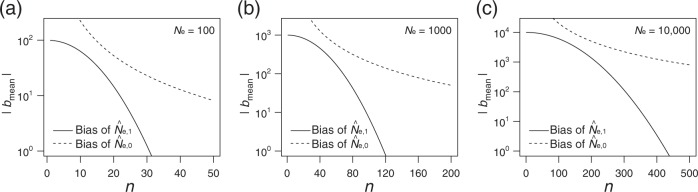
Absolute bias of $\hat N_{{\mathrm{e}},{\mathrm{1}}}$ (represented by a solid line) and $\hat N_{{\mathrm{e}},{\mathrm{0}}}$ (represented by a dotted line) as a function of *n*. **a** *N*_e_ = 100. **b** *N*_e_ = 1000. **c** *N*_e_ = 10,000

First, we evaluated the accuracy of $\hat N_{{\mathrm{e}},{\mathrm{1}}}$ based on its bias (*b*_mean_). For a given *N*_e_, the absolute value of the bias is represented by a solid line in Fig. 3a (*N*_e_ = 100), 3b (*N*_e_ = 1000), and 3c (*N*_e_ = 10,000) as a function of *n*. For comparison, the bias of $\hat N_{{\mathrm{e}},{\mathrm{0}}}$ (see Appendix C) is represented by a dotted line. It is evident that the absolute value of the bias is smaller in $\hat N_{{\mathrm{e}},{\mathrm{1}}}$ than in $\hat N_{{\mathrm{e}},{\mathrm{0}}}$, because the bias of $\hat N_{{\mathrm{e}},{\mathrm{1}}}$ approximately increases with *N*_e_ (Eq. (14)) while the bias of $\hat N_{{\mathrm{e}},{\mathrm{0}}}$ approximately increases with $\hat N_{\mathrm{e}}^2$ (Eq. (S13)). There are remarkable differences between them especially in the situation of small sample size (*n*). Hereafter, we use $\hat N_{{\mathrm{e}},{\mathrm{1}}}$ as the estimator of effective mother size.

### Accuracy and precision of $\hat N_{{\mathrm{e}},{\mathrm{1}}}$

**Fig. 4:**
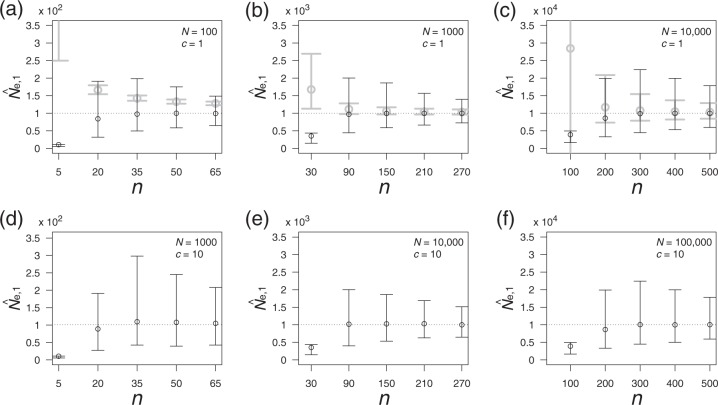
Accuracy and precision of $\hat N_{{\mathrm{e}},{\mathrm{1}}}$ (denoted by black color) and $\hat N_{{\mathrm{e}},{\mathrm{TM}}}$ (denoted by gray color, only appeared in **a**–**c**) as a function of *n*. Open circles represent means with 95% CIs. A dotted line indicates the true value of *N*_e_ which is calculated with given parameters (*N* and *c*). *N*_e_ ≈ 100 in **a** and **d**, *N*_e_ ≈ 1000 in **b** and **e**, and *N*_e_ ≈ 10,000 in **c** and **f**, which are the same value used for calculating $\hat N_{{\mathrm{e}},{\mathrm{TM}}}$. The mean value of $\hat N_{{\mathrm{e}},{\mathrm{TM}}}$ with *n* = 5 in **a** equals 364. For illustrative purposes, only a part of the CI in $\hat N_{{\mathrm{e}},{\mathrm{TM}}}$ is represented in **a** (*n* = 5) and **c** (*n* = 100)

As expected, |*b*_mean_| decreases with *n*, as a larger *n* leads the term $(1 - N_{\mathrm{e}}^{ - 1})^{n_{{\mathrm{pair}}}\,+\,1}$ in *b*_mean_ to vanish more quickly. The requisite sample size (*n*) with a small bias of less than 10% is 22 for *N*_e_ = 100 (|*b*_mean_| < 10; see Fig. 3a), 69 for *N*_e_ = 1000 (|*b*_mean_| < 100; see Fig. 3b), and 216 for *N*_e_ = 10,000 (|*b*_mean_| < 1000; see Fig. 3c). The results of the individual-based model support the above prediction. Figure 4 illustrates the average value of $\hat N_{{\mathrm{e}},{\mathrm{1}}}$ (represented by black open circles) and $\hat N_{{\mathrm{e}},{\mathrm{TM}}}$ (represented by gray open circles; details are provided in the next subsection) with a 95% confidence interval (CI), which is obtained from the individual-based model. As expected, the average value of $\hat N_{{\mathrm{e}},{\mathrm{1}}}$ downwardly deviates from *N*_e_ for a relatively small sample size (*n*) satisfying |*b*_mean_| ≫ 1. As *n* increases, the average value of $\hat N_{{\mathrm{e}},{\mathrm{1}}}$ approaches a true *N*_e_ (represented by a black dotted line in Fig. 4).

Next, we evaluated the precision of $\hat N_{{\mathrm{e}},{\mathrm{1}}}$. As illustrated in Fig. 4, the precision of $\hat N_{{\mathrm{e}},{\mathrm{1}}}$ for a change in *n* behaves in a complex manner. For the investigated parameter set, we determined that the degree of precision holds under different combinations of *N* and *c* if the value of *N*_e_ is fixed (*N*_e_ ≈ *N*/*c* equals 100 in Fig. 4a, d, 1000 in Fig. 4b, e, and 10,000 in Fig. 4c, f); this suggests that the level of uncertainty is roughly determined by *N*_e_. Although the lower limit of the CI monotonically increases with *n*, the upper limit of the CI has a peak at the point at which the average $\hat N_{{\mathrm{e}},{\mathrm{1}}}$ is very close to the true *N*_e_. Near this point, the range of the CI is large, and $\hat N_{{\mathrm{e}},{\mathrm{1}}}$ is asymmetrically distributed with a longer tail on the large side (e.g., *n* = 300 in Fig. 4c, f). As *n* increases beyond this point, the range of the CI decreases, and the shape of the distribution asymptotically becomes symmetric.

### Comparison of $\hat N_{{\mathrm{e}},{\mathrm{1}}}$ with $\hat N_{{\mathrm{e}},{\mathrm{TM}}}$

As shown in Fig. 4a–c, $\hat N_{{\mathrm{e}},{\mathrm{1}}}$ is much more accurate than $\hat N_{{\mathrm{e}},{\mathrm{TM}}}$ for most of the sample sizes (the number of loci is 500 and the interval generation of sampling is ten; see details in the “Theory” section). In addition, with a relatively small sample size, $\hat N_{{\mathrm{e}},{\mathrm{1}}}$ is much more precise than $\hat N_{{\mathrm{e}},{\mathrm{TM}}}$. It is generally known that, when *N*_e_ is relatively large and sample size is relatively small, the estimated value by the temporal method becomes flawed because the sampling variance overshadows the magnitude of genetic drift that determines the accuracy in the temporal method (Nei and Tajima 1981). Such a situation was confirmed in our setting, as an appearance of negative $\hat N_{{\mathrm{e}},{\mathrm{TM}}}$. (*N*_e_ = 10,000 and *n* = 100; see Fig. 4c). Even when *N*_e_ is relatively small, the accuracy of $\hat N_{{\mathrm{e}},{\mathrm{1}}}$ is higher than that of $\hat N_{{\mathrm{e}},{\mathrm{TM}}}$ (*N*_e_ = 100; see Fig. 4a). Together with the theoretical predictions (as shown in Fig. 3), we conclude that the high performance of our method is due to the bias reduction of the estimator, especially in a relatively small sample size.

### Accuracy of $\hat v$ and $\widehat {cv}$

**Fig. 5:**
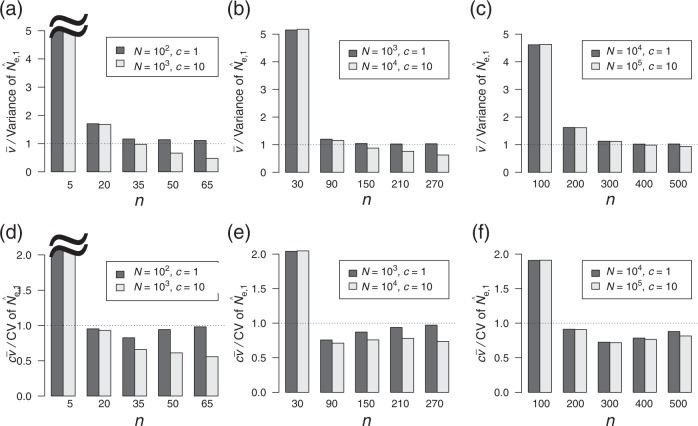
**a**–**c** Ratio of the average $\hat v$ to the variance of $\hat N_{{\mathrm{e}},{\mathrm{1}}}$ as a function of *n*. **d**–**f** Ratio of the average $\widehat {cv}$ to the coefficient of variation of $\hat N_{{\mathrm{e}},{\mathrm{1}}}$ as a function of *n*. The value of parameters (*N* and *c*) is indicated in the legend. **a**, **d** *N*_e_ ≈ 100. **b**, **e** *N*_e_ ≈ 1000. **c**, **f** *N*_e_ ≈ 10,000

We then evaluated the accuracy of $\hat v$. Theoretically, the bias of $\hat v$ (*b*_var_) was determined to have a peak at a certain value of *n*, as illustrated in Fig. S3 (Supplementary Information). Figure 5a–c presents the ratio of the average $\hat v$ to the variance of $\hat N_{{\mathrm{e}},{\mathrm{1}}}$ for different combinations of (*N*, *c*) with fixed *N*_e_, which is obtained from the individual-based model. If the ratio is close to 1, $\hat v$ is deemed an estimator of unbiasedness. When *n* approaches zero, the ratio becomes inflated (e.g., *n* = 5 in Fig. 5a) although *b*_var_ also approaches zero (Fig. S3). This inconsistency for a small *n* (i.e., overestimation) may result from the bias of $\hat N_{{\mathrm{e}},{\mathrm{1}}}$. As *n* increases, the ratio approaches 1 when *c* = 1 but less than 1 when *c* > 1 (*c* = 10 in Fig. 5a–c), suggesting that the property of unbiasedness holds only under the Poisson variance; however, the degree of this bias is not very high for a relatively large *N*_e_ (*c* = 10 in Fig. 5c). In other words, the accuracy of $\hat v$ is not solely determined by the level of *N*_e_. This inconsistency (i.e., underestimation) may result from the assumption that the correlation between pairs can be ignored and thus that the number of HS pairs in the sample follows a binomial distribution (Eq. (8)).

Finally, we evaluated the accuracy of $\widehat {cv}$. Figure 5d–f illustrates the ratio of the average $\widehat {cv}$ to the coefficient of variation of $\hat N_{{\mathrm{e}},{\mathrm{1}}}$, which is obtained from the individual-based model. As expected, the property of the estimator is similar to that of $\hat v$, as $\widehat {cv}$ is defined by using $\hat v$ (Eq. (17)). The ratio becomes inflated for small *n*; as *n* increases, the ratio approaches 1 when *c* = 1 but is <1 when *c* > 1 (i.e., underestimation); however, the relationship between the degree of bias and the level of *N*_e_ is unclear.

## Discussion

We theoretically developed a nearly unbiased estimator of the number of effective mothers in a population ($\hat N_{{\mathrm{e}},{\mathrm{1}}}$), the estimator of its variance ($\hat v$), and its coefficient of variation ($\widehat {cv}$), which are based on the known MHS relationships found within a single cohort. The performance of the estimators (accuracy and precision) was quantitatively evaluated by running an individual-based model. Our modeling framework allows for two types of reproductive variation; variance of the average offspring number per mother (parental variation) and variance of the offspring number across mothers with the same reproductive potential (nonparental variation). The former is related to age- or size-dependent reproductive potential, whereas the latter is related to family-correlated survival, both of which can result in a skewed distribution of offspring number. These two effects are summarized into one parameter (*c*) in the framework. Our estimators can be calculated from sample size (*n*) and the observed number of MHS pairs (*H*_obs_) and do not require other parameters, such as adult mother size (*N*) or the degree of overdispersed reproduction (*c*). The rationale for this is that the observed number of MHS pairs contains information about these parameters.

To estimate the number of effective mothers (*N*_e_), our theoretical results provide guidance for a sample size to ensure the required accuracy and precision, especially if the order of the number of effective mothers is approximately known. For example, when the effective number of mothers is within 10^2^–10^3^, sampling 50 offspring falls within the range of accuracy of the estimation with a 0–30% bias (Eq. (14) and Fig. 3). Even if there is no information about the effective number of mothers, the coefficient of variation of the estimated number can be estimated ($\widehat {cv}$) when the sample size is above a given level (Fig. 5c, d). Although the estimator of the variation of the number of mothers ($\hat v$) is relatively accurate for the investigated parameter set (Fig. 5a, b), the present estimator of the coefficient of variation is systematically biased; thus, improvements in accuracy are left for future research.

Our modeling framework is presented in the context of the sibship assignment (SA) method, which defined a kinship-oriented estimation of effective population size (Wang 2009; Waples and Waples 2011; Ko and Nielsen 2019). The original theory of the SA method was developed by Wang (2009), and it can perform the estimation of effective population size from HS and FS probabilities, which are calculated by the number of HS and FS pairs in a sample. Wang’s estimator reduces to the inverse of the frequency of HS pairs in a sample, which corresponds to $\hat N_{{\mathrm{e}},{\mathrm{0}}}$ (Eq. (11)). Our proposed estimator $\hat N_{{\mathrm{e}},{\mathrm{1}}}$ (Eq. (13)) is more accurate than $\hat N_{{\mathrm{e}},{\mathrm{0}}}$, because the bias of $\hat N_{{\mathrm{e}},{\mathrm{1}}}$ approximately increases with *N*_e_ (Eq. (14)) while the bias of $\hat N_{{\mathrm{e}},{\mathrm{0}}}$ approximately increases with $\hat N_{\mathrm{e}}^2$ (Eq. (S13)). There are remarkable differences between them specially for small sample sizes, as shown in Fig. 3. In this study, we analytically obtained nearly unbiased estimators ($\hat N_{{\mathrm{e}},{\mathrm{1}}}$, $\hat v$, and $\widehat {cv}$), although their application is limited to the estimation of effective mother size and the case in which MHS can be perfectly distinguished from PHS and other relatives. The latter limitation may be overcome to some extent by the use of a hypervariable region in the mitochondrial genome and/or sex-linked markers. It should be noted that genetic differentiation between maternal and paternal relatives is a general problem with pedigree reconstruction (Huisman 2017; Hillary et al. 2018). Therefore, incorporating the uncertainty of differentiation or modifying the theory with the use of HS (not MHS) remains a task for future research.

As a first step in developing unbiased estimators of *N*_e_, we examined a relatively simple situation and ignored the complex but important features required in practical scenarios, including errors associated with kinship detection and nonrandom sampling. In general, SAs are biased when only limited molecular information is available (e.g., small number or poor quality of genetic markers), and direction of the biases depends on kinship detection algorithm and how to incorporate prior knowledge into the algorithm. Uncertainties of the proposed estimator of *N*_e_ due to limited molecular data could be assessed if an algorithm for SAs is incorporated into our framework. It is expected that if nonrandom sampling is caused by a family-correlated sampling scheme, the effective mother size is underestimated because MHS pairs are more likely to be sampled with this sampling scheme than with random sampling. To reduce this bias, the sampling time and location should be varied, or sampling at an early life stage after hatching should be avoided; this may reduce the effect of family-correlated movement that is not addressed in the current theoretical framework.

Contemporary effective population size can provide not only an understanding of genetic health but also an indication of adult size. If the effect of overdispersion *c* is invariant across years, $\hat N_{{\mathrm{e}},{\mathrm{1}}}$ may behave as an index of the number of mothers per year, making it possible to determine the temporal trends, since Eq. (13) can be rewritten as

18 $$\widehat {\left( {\frac{{N - 1}}{c}} \right)} = \hat N_{{\mathrm{e}},{\mathrm{1}}} - 1.$$

In this case, the proposed index becomes highly informative, particularly for integrating stock assessment in fisheries management using many types of data (e.g., catch data and abundance index data); this leads to more accurate estimation due to the use of fishery-independent data (Ovenden et al. 2015). Recently, Akita (2018) developed a summary statistic that indicates the degree of overdispersion; this statistic is based on the number of MHS pairs and mother–offspring pairs in the sample. The temporal trend of this statistic provides information on whether *c* is invariant across the years and thus provides criteria for determining whether $\hat N_{{\mathrm{e}},{\mathrm{1}}}$ behaves as an index of the number of mothers in a population. It should be noted that, if census size is independently obtained, combined with the estimated effective size, we can estimate the magnitude of reproductive variance and potentially the parameter of nonparental variation (i.e., *ϕ*), which is generally difficult to obtain and can provide unavailable insights into the underlying ecological processes.

Finally, we note the theoretical connection of our results to the ratio of effective mother size to census size, *N*_e_/*N*. A number of studies have demonstrated that the ratio of the effective size to the census size (including fathers) in high-fecundity marine species is estimated to fall within 10^−3^–10^−6^ (Hauser and Carvalho 2008). In our derivation, *N*_e_/*N* is approximately equal to 1/*c*. If there is only parental variation (i.e., *c* = $${\Bbb E}$$[*λ*^2^]/$${\Bbb E}$$[*λ*]^2^ = $${\Bbb C}$$$${\Bbb V}$$[*λ*]^2^ + 1), *c* cannot have a large value; thus, the ratio cannot become very small (e.g., <10^−3^). This theoretical consideration suggests a dominant contribution of nonparental variation to a very small *N*_e_/*N*, which is consistent with the result in Waples (2016).

### Supplementary information

Supplemental_Material

## A. Probability density function and its moment of *λ*

As noted in the main text, our modeling framework does not require the specific form of *f*(*λ*); instead, it only requires the ratio of the second moment to the squared first moment (${\Bbb E}$[*λ*^2^]/${\Bbb E}$[*λ*]^2^). However, the specific form is required for illustrative purposes (Fig. 2a) and for the evaluation of the theoretical results (i.e., calculating the moment or running the individual-based model). Here, we model an age-structured fish population, which serves as a representative example, demonstrating both parental and nonparental variations.

Suppose that the mean fecundity of a mother depends on her age. Let *λ*_*a*_ be mean fecundity, which is a function of age (denoted by *a*). The moment can be described as ${\Bbb E}[\lambda ^m] = \mathop {\sum}_{a = 0}^{a_{{\mathrm{max}}}} {\lambda _a^m} h_{{\mathrm{mat}}}(a)$, where *h*_mat_(*a*) is the frequency of mature mothers at a given age, and *a*_max_ is the maximum age. Thus, we can numerically obtain the moment from *λ*_*a*_ and *h*_mat_(*a*).

For marine species with a type-III survivorship curve, it is generally assumed that individual fecundity is proportional to weight. Using the von Bertalanffy growth equation for body weight, *λ*_*a*_ is explicitly described as a function of age as follows (identical to Eq. (4)):

$$\lambda _a \propto (1 - {\mathrm{exp}}[ - \kappa (a - a_0)])^\beta ,$$

where *κ*, *a*_0_, and *β* are conventionally used parameters in the von Bertalanffy equation and represent the growth rate, the adjuster of the equation for the initial size of the animal, and the allometric growth parameter, respectively. For obtaining a specific value of *λ*, a coefficient value of 10 multiplied by the right-hand side of Eq. (4) was used when running the individual-based model.

The frequency of mature mothers at a given age can be written as follows:

S1 $$h_{{\mathrm{mat}}}(a) \propto h(a)Q(a),$$

satisfying $\mathop {\sum}_{a = 0}^{a_{{\mathrm{max}}}} {h_{{\mathrm{mat}}}} (a) = 1$, where *h*(*a*) and *Q*(*a*) represent the frequency and maturity at a given age, respectively. Although *f*(*a*) is affected by historical population dynamics and age-dependent survival, for simplicity, the mortality rate is assumed to be constant (i.e., age independent):

S2 $$h(a) \propto \left\{ {\begin{array}{*{20}{l}} {S^a} \hfill \\ 0 \hfill \end{array}\begin{array}{*{20}{l}} {{\mathrm{if}}\;a\, {<} \,a_{{\mathrm{max}}}} \hfill \\ {{\mathrm{if}}\;a = a_{{\mathrm{max}}}} \hfill \end{array}} \right.,$$

where *S* is survival probability. Maturity at age (*Q*(*a*)) is assumed to be a knife-edge function, given by

S3 $$Q(a) = \left\{ {\begin{array}{*{20}{l}} 1 \hfill \\ 0 \hfill \end{array}\begin{array}{*{20}{l}} {{\mathrm{if}}\;a \ge a_{{\mathrm{mat}}}} \hfill \\ {{\mathrm{otherwise}}} \hfill \end{array}} \right.,$$

where *a*_mat_ is the mature age.

For calculating ${\Bbb E}$[*λ*^2^]/${\Bbb E}$[*λ*]^2^, the required parameter set is (*a*_max_, *κ*,*a*_0_, *β*, *S*, *a*_mat_). In this paper, for the purpose of representation, we fixed the value of several parameters as follows: *a*_max_ = 20, *κ* = 0.3, *a*_0_ = 0, *S* = 0.5 and *a*_mat_ = 0. In addition, we selected parameter value *c* (=(1 + *ϕ*^−1^)${\Bbb E}$[*λ*^2^]/${\Bbb E}$[*λ*]^2^) to be 1 and 10 for comparison with the results in the main text, which are derived from the parameter set (*ϕ*, *β*) = (1000, 0.0009) and (0.1302, 0.9), respectively.

Finally, we provide the specific forms of *f*(*λ*) and Pr[*k*], which are presented in the main text (Fig. 2). When *λ*_*a*_ and *h*_mat_(*a*) are obtained, *f*(*λ*) is given by

S4 $$f(\lambda ) = \left\{ {\begin{array}{*{20}{l}} {h_{{\mathrm{mat}}}(a)} \hfill \\ 0 \hfill \end{array}\begin{array}{*{20}{l}} {{\mathrm{if}}\;\lambda = \lambda _a} \hfill \\ {{\mathrm{otherwise}}} \hfill \end{array}} \right..$$

Using Eqs. (1) and (S 4), we can obtain the specific form of the marginal distribution of *k* by

S5 $${\mathrm{Pr}}[k] = \mathop {\sum}\limits_\lambda {{\mathrm{Pr}}} [k|\lambda ]f(\lambda ).$$

## B. Probability that two offspring share an MHS relationship

Given the realized number of offspring *k*_1_, *k*_2_, …, *k*_*N*_, the probability that two randomly selected offspring are born to mother *i* is $k_i/\!\mathop {\sum}_{j\,=\,1}^N {k_j}\,\times\,(k_i - 1)/(\mathop {\sum}_{j\,=\,1}^N {k_j} - 1)$. Thus, the conditional probability that two offspring share an MHS relationship with an arbitrary mother is

$$\pi |k_1, \ldots \!,\,k_N = \mathop {\sum}_{i = 1}^N {\frac{{k_i(k_i - 1)}}{{(\mathop {\sum}_{j = 1}^N {k_j} )(\mathop {\sum}_{j = 1}^N {k_j} - 1)}}}$$

S6 $$= \frac{{\mathop {\sum}_{i = 1}^N {k_i^2} - \mathop {\sum}_{i = 1}^N {k_i} }}{{(\mathop {\sum}_{j = 1}^N {k_j} )^2 - \mathop {\sum}_{j = 1}^N {k_j} }}.$$

It should be noted that *k*_*i*_ is a random variable followed by a negative binomial distribution (Eq. (1)), in which the parameter of the distribution, *λ*_*i*_, is also a random variable followed by an arbitral function *f*(*λ*). By taking the expectation over the distribution of offspring number, the conditional probability is given by

$$\pi |\lambda _1, \ldots\!,\lambda _N = {\Bbb E}\left[ {\pi |k_1, \ldots\!,k_N} \right]$$

$$\approx \frac{{\mathop {\sum}_{i = 1}^N {\Bbb E} [k_i^2|\lambda _i] - \mathop {\sum}_{i = 1}^N {\Bbb E} [k_i|\lambda _i]}}{{{\Bbb E}[(\mathop {\sum}_{j = 1}^N {k_j} )^2|\lambda _1, \ldots\!,\lambda _N] - \mathop {\sum}_{j = 1}^N {\Bbb E} [k_j|\lambda _j]}}$$

S7 $$= \frac{{\mathop {\sum}_{i\,=\,1}^N {(1\,+\,\phi ^{ - 1})} \lambda _i^2}}{{\mathop {\sum}_{i\,=\,1}^N {(1\,+\,\phi ^{ - 1})} \lambda _i^2\,+\,2\mathop {\sum}_{i > j} {\lambda _i} \lambda _j}}.$$

From the first to the second line, we use the approximation that $${\Bbb E}$$[*g*_1_(*k*)/*g*_2_(*k*)]$$\approx$$$${\Bbb E}$$[*g*_1_(*k*)]/$${\Bbb E}$$[*g*_2_(*k*)]. The expectations are averaged over *k* or *k*^2^, conditional on *λ*. By taking the expectation over *λ* and applying a similar approximation, the unconditional probability is given by

$$\pi = {\Bbb E}[\pi |\lambda _1, \ldots \!,\lambda _N]$$

S8 $$\approx \frac{{(1\,+\,\phi ^{ - 1}){\Bbb E}[\lambda ^2]}}{{(1\,+\,\phi ^{ - 1}){\Bbb E}[\lambda ^2]\,+\,(N - 1)({\Bbb E}[\lambda ])^2}}.$$

This provides the formulation described in Eq. (3). In computing the expectation, we remove the subscript (*i* or *j*) because *λ* is independent and identically distributed.

## C. Properties of moment estimator of *N*

Here, we demonstrate that $\hat N_{{\mathrm{e}},{\mathrm{0}}}$ in Eq. (11) is the maximum likelihood estimator and that $\hat N_{{\mathrm{e}},{\mathrm{0}}}$ is upwardly biased, especially when *n* is small. Let *L* be the likelihood of the distribution of *H* (Eq. (8)). Given the observation (i.e., *H*_obs_), the partial derivative of the log-likelihood with respect to *π* is given by

S9 $$\frac{{\partial {\mathrm{ln}}L}}{{\partial \pi }} \propto \frac{{H_{{\mathrm{obs}}}n_{{\mathrm{pair}}}}}{\pi } - \frac{{(n_{{\mathrm{pair}}} - H_{{\mathrm{obs}}})n_{{\mathrm{pair}}}}}{{1 - \pi }},$$

leading to the maximum likelihood estimator of *π*:

S10 $$\hat \pi = \frac{{H_{{\mathrm{obs}}}}}{{n_{{\mathrm{pair}}}}},$$

where $\hat \pi$ satisfies $(\partial {\mathrm{ln}}L/\partial \pi )|_{\pi = \hat \pi } = 0$. Substituting Eq. (3) into Eq. (S10), we can obtain the estimator ($\hat N_{{\mathrm{e}},{\mathrm{0}}}$) described in Eq. (11).

Consider the bias of $\hat N_0$ defined by ${\Bbb E}[\hat N_{{\mathrm{e}},{\mathrm{0}}}] - N_{\mathrm{e}}$. We set the following equations:

$$g(H) = \hat N_{{\mathrm{e}},{\mathrm{0}}}$$

S11 $$= \frac{{n_{{\mathrm{pair}}}}}{H},$$

and

$$g(\mu ) = N_{\mathrm{e}}$$

S12 $$= \frac{{n_{{\mathrm{pair}}}}}{\mu },$$

where $\mu = {\Bbb E}[H]$. Using Eqs. (9) and (10), the bias is given by

$${\Bbb E}[g(H) - g(\mu )] \approx \frac{1}{2}{\Bbb E}[g\prime\prime (H)(H - \mu )^2]$$

$$= \frac{1}{2}\frac{{2n_{{\mathrm{pair}}}}}{{\mu ^3}}{\Bbb V}[H]$$

S13 $$\approx \frac{{N_{\mathrm{e}}^2}}{{n_{{\mathrm{pair}}}}},$$

where a quadratic approximation for *g* centered at *μ* and ${\Bbb V}$[*H*] ≈ ${\Bbb E}$[*H*] is used. The value of the right-hand side of Eq. (S13) is illustrated in Fig. 3a, b as the bias of $\hat N_{{\mathrm{e}},{\mathrm{0}}}$.

## D. Derivation of nearly unbiased estimator of *N*_e_

We consider the following

$${\Bbb E}\left[ {\frac{1}{{H\,+\,1}}} \right]\,=\,\mathop {\sum}\limits_{h\,=\,0}^{n_{{\mathrm{pair}}}} {\frac{1}{{h\,+\,1}}} {\mathrm{Pr}}[h|n_{{\mathrm{pair}}}]$$

$$= \frac{1}{{(n_{{\mathrm{pair}}}\,+\,1)\pi }}\mathop {\sum}\limits_{h{\prime}\,=\,1}^{n_{{\mathrm{pair}}}\,+\,1} {{\mathrm{Pr}}} [h\prime |n_{{\mathrm{pair}}} + 1]$$

$$= \frac{1}{{(n_{{\mathrm{pair}}} + 1)\pi }}(1 - {\mathrm{Pr}}[h\prime = 0|n_{{\mathrm{pair}}} + 1])$$

S14 $$= \frac{{1 - (1 - \pi )^{n_{{\mathrm{pair}}}\,+\,1}}}{{(n_{{\mathrm{pair}}}\,+\,1)\pi }},$$

assuming the binomial form of *H* (Eq. (8)). Equation (S14) is not directly applied for the derivation of the estimator of *N*_e_ due to the complex formulation. Thus, we simplify the formulation as follows:

S15 $${\Bbb E}\left[ {\frac{1}{{H\,+\,1}}} \right] \approx \frac{1}{{(n_{{\mathrm{pair}}}\,+\,1)\pi }},$$

assuming that

S16 $$(1 - \pi )^{n_{{\mathrm{pair}}}\,+\,1} \approx 0.$$

This simplification deviates from the prediction by Eq. (S14) when *n* is relatively small. Replacing ${\Bbb E}$[1/(*H* + 1)] by 1/(*H*_obs_ + 1) in the left-hand side of Eq. (S15), we can obtain the estimator ($\hat N_{{\mathrm{e}},{\mathrm{1}}}$) described in Eq. (13).

For the evaluation of $\hat N_{{\mathrm{e}},{\mathrm{1}}}$, the bias is calculated. ${\Bbb E}[\hat N_{{\mathrm{e}},{\mathrm{1}}}]$ is required for the calculation and given by

$${\Bbb E}[\hat N_{{\mathrm{e}},{\mathrm{1}}}] = (n_{{\mathrm{pair}}}\,+\,1){\Bbb E}\left[ {\frac{1}{{H\,+\,1}}} \right]$$

S17 $$= N_{\mathrm{e}} - N_{\mathrm{e}}(1 - N_{\mathrm{e}}^{ - 1})^{n_{{\mathrm{pair}}} +\,1},$$

where the relationship in Eq. (S14) is used. This provides the formulation of the bias, as described in Eq. (14).

## E. Derivation of estimator of variance of $\hat N_{{\mathrm{e}},{\mathrm{1}}}$

Let *v* be the estimator of the variance of $\hat N_{{\mathrm{e}},{\mathrm{1}}}$. It is desirable for *v* to be defined such that the bias (denoted *b*_var_) is reasonably small. From Eq. (13), the variance of $\hat N_{{\mathrm{e}},{\mathrm{1}}}$ is given by

$${\Bbb V}[\hat N_{{\mathrm{e}},{\mathrm{1}}}] = (n_{{\mathrm{pair}}} + 1)^2{\Bbb V}\left[ {\frac{1}{{H + 1}}} \right]$$

$$= (n_{{\mathrm{pair}}}\,+\,1)^2\left( {{\Bbb E}\left[ {\frac{1}{{(H\,+\,1)^2}}} \right] - {\Bbb E}\left[ {\frac{1}{{(H\,+\,1)}}} \right]^2} \right)$$

S18 $$= {\Bbb E}\left[ {\frac{{(n_{{\mathrm{pair}}}\,+\,1)^2}}{{(H\,+\,1)^2}}} \right] - N_{\mathrm{e}}^2(1 - (1 - N_{\mathrm{e}}^{ - 1})^{n_{{\mathrm{pair}}}\,+\,1})^2$$

where the term ${\Bbb E}$[1/(*H* + 1)] is calculated from the relationship in Eq. (S14). Roughly speaking, ${\Bbb V}[\hat N_{{\mathrm{e}},{\mathrm{1}}}]$ is dominated by two terms when *n*_pair_ is relatively large: ${\Bbb E}$[(*n*_pair_ + 1)^2^/(*H* + 1)^2^] and $- \hat N_{\mathrm{e}}^2$. Thus, it is expected that ${\Bbb E}$[*v*] includes both terms for a reasonably small bias. We propose the following formulation for *v*:

$$v = \frac{{(n_{{\mathrm{pair}}} + 1)(n_{{\mathrm{pair}}} - H)}}{{(H + 1)^2(H + 2)}}$$

S19 $$= \frac{{(n_{{\mathrm{pair}}} + 1)^2}}{{(H + 1)^2}} - \frac{{(n_{{\mathrm{pair}}} + 1)(n_{{\mathrm{pair}}} + 2)}}{{(H + 1)(H + 2)}}.$$

The expectation of the second term in Eq. (S19) is given by

$${\Bbb E}\left[ {\frac{{(n_{{\mathrm{pair}}} + 1)(n_{{\mathrm{pair}}} + 2)}}{{(H + 1)(H + 2)}}} \right]$$

$$= (n_{{\mathrm{pair}}}\,+\,1)(n_{{\mathrm{pair}}}\,+\,2)\mathop {\sum}\limits_{h\,=\,0}^{n_{{\mathrm{pair}}}} {\frac{1}{{(h\,+\,1)(h\,+\,2)}}} {\mathrm{Pr}}[h|n_{{\mathrm{pair}}}]$$

$$= \frac{1}{{\pi ^2}}\mathop {\sum}\limits_{h{\prime} = 2}^{n_{{\mathrm{pair}}} + 2} {{\mathrm{Pr}}} [h\prime |n_{{\mathrm{pair}}} + 2]$$

$$= N_{\mathrm{e}}^2(1 - {\mathrm{Pr}}[h\prime = 0|n_{{\mathrm{pair}}} + 2] - {\mathrm{Pr}}[h\prime = 1|n_{{\mathrm{pair}}} + 2])$$

S20 $$= N_{\mathrm{e}}^2(1 - (1 - N_{\mathrm{e}}^{ - 1})^{n_{{\mathrm{pair}}} + 2} - (n_{{\mathrm{pair}}} + 2)N_{\mathrm{e}}^{ - 1}(1 - N_{\mathrm{e}}^{ - 1})^{n_{{\mathrm{pair}}} + 1}),$$

leading to a relatively small *b*_var_ when *n*_pair_ is large, which is described in Eq. (16).
