# Supplementary material for: Nearly unbiased estimator of contemporary effective mother size using within-cohort maternal sibling pairs incorporating parental and nonparental reproductive variations
Source: Heredity (Edinb). 2019 Oct 2;124(2):299–312. doi: 10.1038/s41437-019-0271-6 (PMC6972959; doi:10.1038/s41437-019-0271-6)
Supplement: Supplementary file 1 — Supplemental_Material [file 41437_2019_271_MOESM1_ESM.pdf]

Supplementary Information

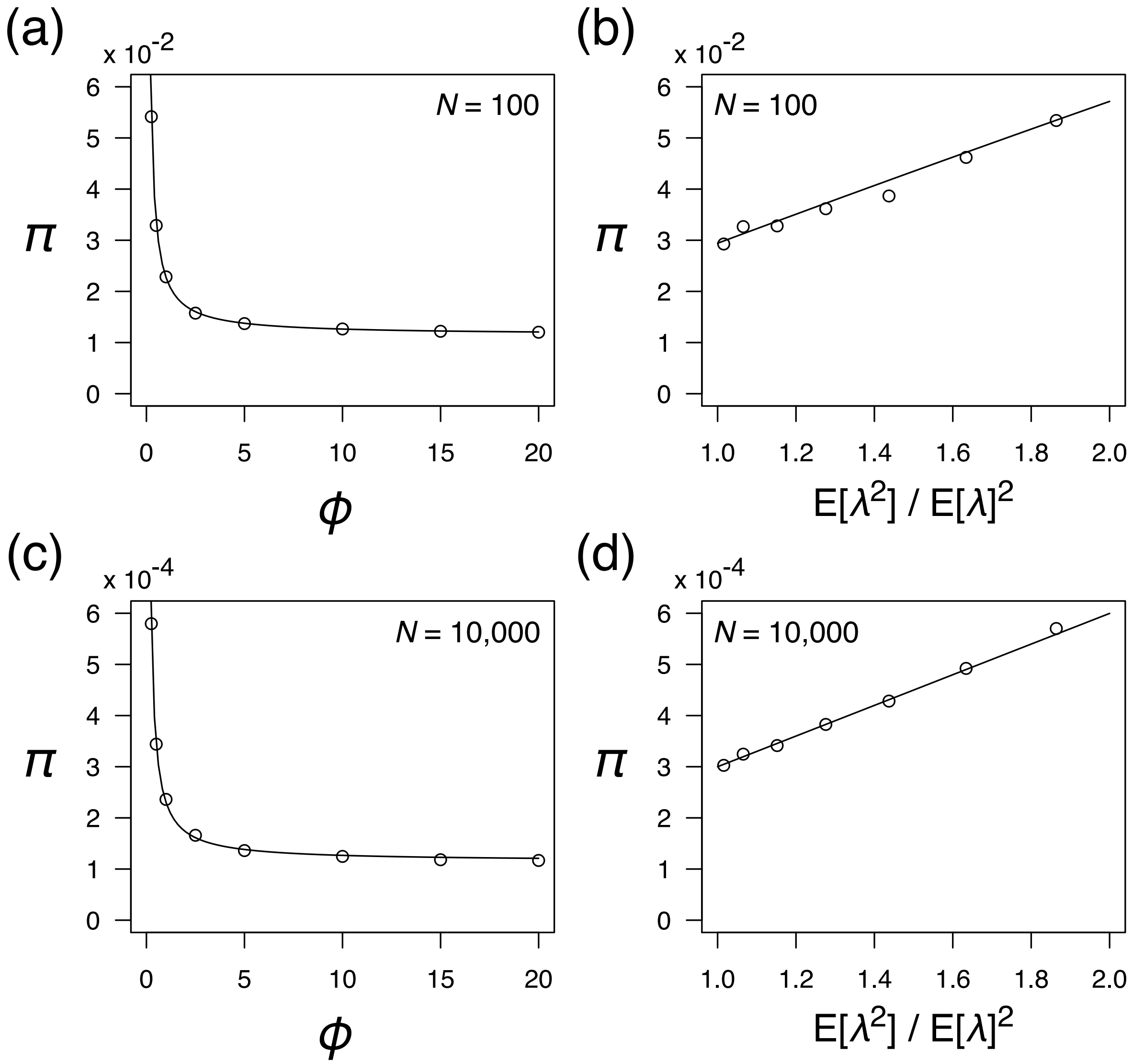

Figure S1: Accuracy of approximation for π as a function of (a), (c) ϕ and (b), (d) $\mathbb{E}[\lambda^2]/\mathbb{E}[\lambda]^2$. The thin line represents the approximated values (Eq. 3), and the points represent the simulated values from 10,000,000 replications. (b), (d) $\mathbb{E}[\lambda^2]/\mathbb{E}[\lambda]^2$ is calculated from the distribution of λ (see Appendix A) with $\beta = 0.3, 0.6, 0.9, 1.2, 1.5, 1.8$, and 2.1. (a) $\beta = 0.9$. (b) $\phi = 0.5$. The definition of parameter β is described in Appendix A.

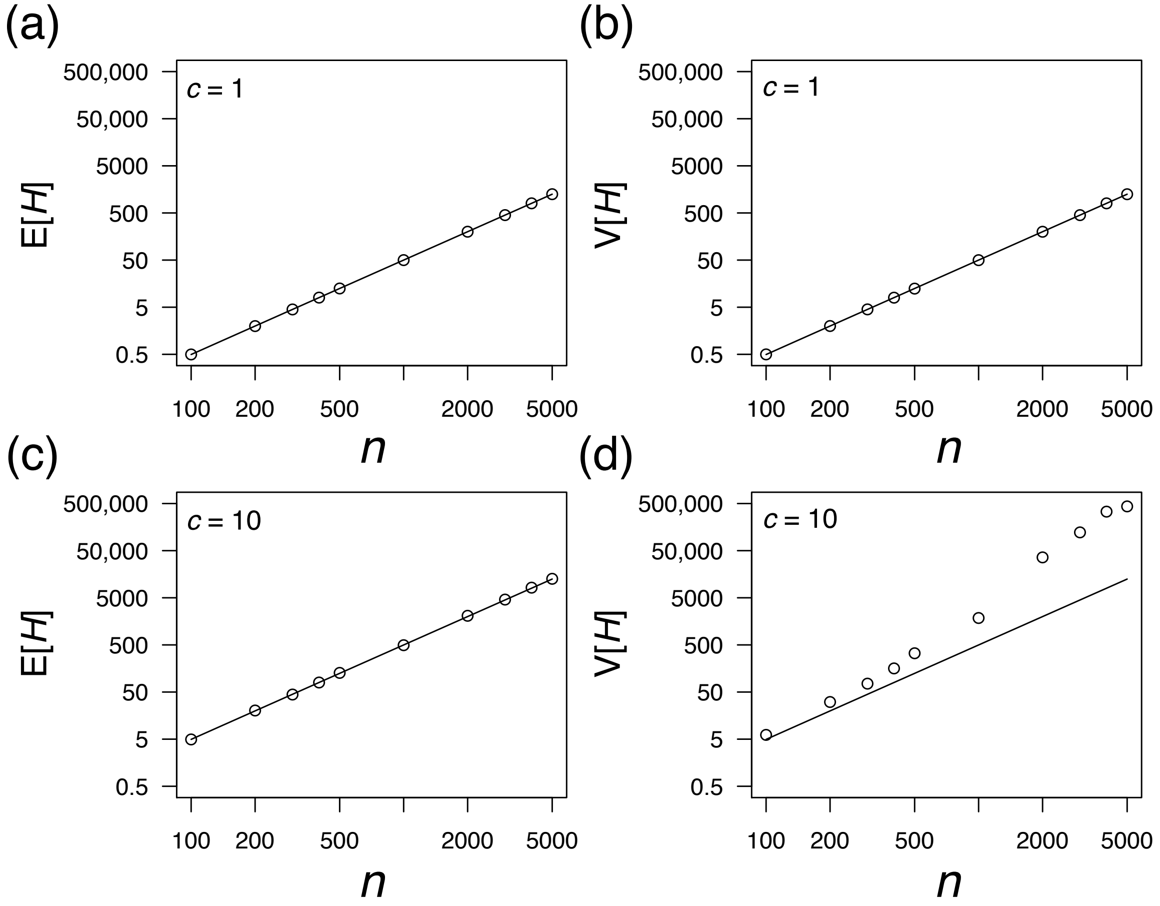

Figure S2: Accuracy of theoretical prediction for (a), (b) $\mathbb{E}[H]$ and (c), (d) $\mathbb{V}[H]$ as a function of n. Thin lines indicate theoretical values (see Eq.s 7 and 8) and points are obtained from simulated data (1,000,000 replications). The value of c is indicated in the legend. $N = 10{,}000$. Both the x-axis and y-axis are log-scale.

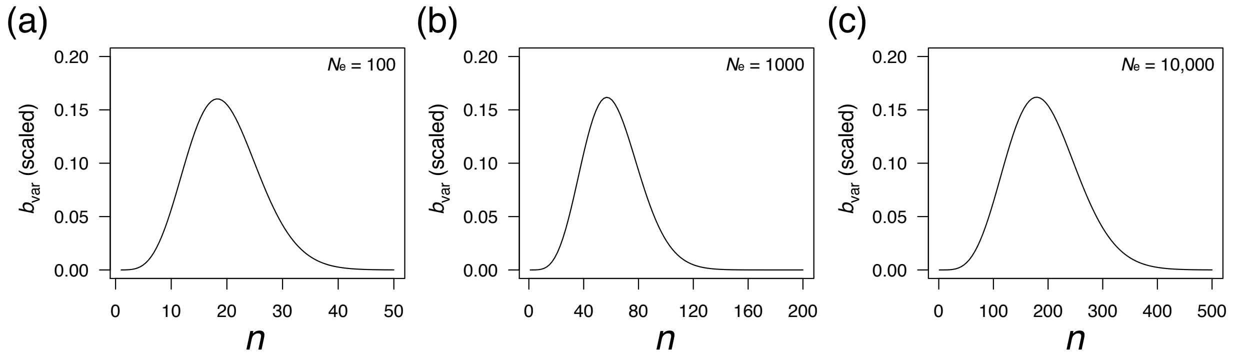

Figure S3: Bias of $\mathbb{V}[\hat{N}_{e,1}]$ as a function of n. The value of the bias is scaled by N_e^2. (a) $N_e = 100$. (b) $N_e = 1000$. (c) $N_e = 10{,}000$.
